# Anti-Inflammatory Mechanisms of Dietary Flavones: Tapping into Nature to Control Chronic Inflammation in Obesity and Cancer

**DOI:** 10.3390/ijms232415753

**Published:** 2022-12-12

**Authors:** Anastasia Kariagina, Andrea I. Doseff

**Affiliations:** 1 Department of Microbiology and Molecular Genetics, Michigan State University, East Lansing, MI 48824, USA; 2 Department of Physiology, Michigan State University, East Lansing, MI 48824, USA; 3 Department of Pharmacology and Toxicology, Michigan State University, East Lansing, MI 48824, USA

**Keywords:** flavonoids, monocytes, macrophages, transcription factors, target identification, cytokines

## Abstract

Flavones are natural phytochemicals broadly distributed in our diet. Their anti-inflammatory properties provide unique opportunities to control the innate immune system and inflammation. Here, we review the role of flavones in chronic inflammation with an emphasis on their impact on the molecular mechanisms underlying inflammatory diseases including obesity and cancer. Flavones can influence the innate immune cell repertoire restoring the immune landscape. Flavones impinge on NF-κB, STAT, COX-2, or NLRP3 inflammasome pathways reestablishing immune homeostasis. Devoid of adverse side effects, flavones could present alternative opportunities for the treatment and prevention of chronic inflammation that contributes to obesity and cancer.

## 1. Introduction

Chronic inflammatory diseases (CI), including obesity and cancer, are reaching epidemic levels affecting more than 60% of the United States population [1]. CID also have a profound socioeconomic impact representing 75% of the total healthcare spending of ~$2 trillion in the United States in 2018 [2]. Importantly, CID are more prevalent in socioeconomically deprived groups. For example, African American women are 70% more likely to be obese compared to white women [3]. Similarly, African American women have a 42% greater risk of cancer-related death and significantly lower rates of survival (78.9%) than non-Hispanic white females (88.6%) [4,5]. Overall, cancer incidence is 20% greater among residents of socioeconomically poor localities [6]. Thus, it has been estimated that the elimination of socioeconomic disparities alone might prevent up to 34% of all cancer-related deaths in the United States [7].

The innate immune system plays a key role in CID. Chronic inflammation is associated with carcinogenesis and obesity [8,9,10]. Key cellular components of the innate immune system include myeloid-derived suppressor cells (MDSC), dendritic cells (DC), monocytes, and macrophages, which regulate the tumor and adipose tissue (AT) microenvironments [11,12,13]. Dysregulated inflammation, characteristic of CID, is usually controlled by non-steroidal anti-inflammatory drugs (NSAIDs). The consumption of NSAIDs has been correlated with decreased risks of breast, bladder, lung, colorectal, prostate, and cervical cancers [14]. Unfortunately, long-term intake of NSAIDs has been associated with adverse effects such as cardiotoxicity and gastrointestinal damage [15]. Therefore, there is a pressing need to identify new approaches that can regulate inflammation with minimal off-target effects.

In that regard, the use of plant nutraceuticals has attracted great attention in regulating inflammation without adverse effects. Among them, the flavones, a subgroup of flavonoids with potent anti-inflammatory, anti-obesogenic, and anti-cancer activities, has gained special consideration [16]. Here, we review the latest advances in the mechanistic role of flavones in the regulation of the innate immune response, with an emphasis on the key pathways that regulate inflammation including NF-κB, STAT, and Nrf2, and their potential impact on CID such as obesity and cancer [17,18,19,20]. The potential of flavones as regulators of the immune response, accompanied by their lack of adverse effects, provides new opportunities for tapping into these natural resources to tackle CID.

## 2. Flavones: Structural Characteristics and Distribution

**Figure 1:**
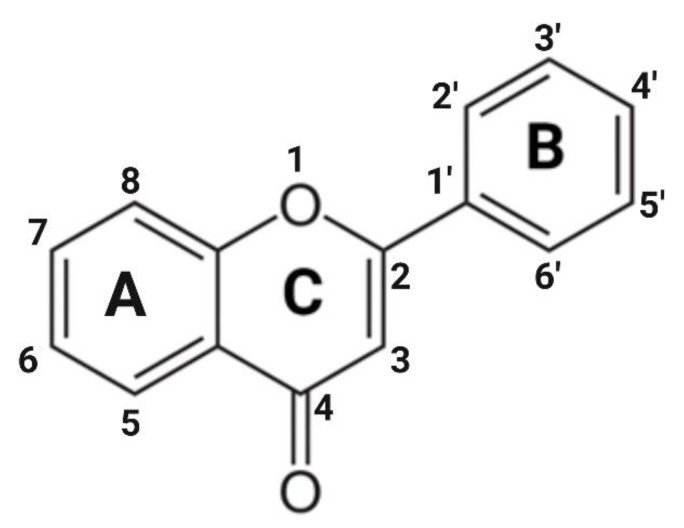
The basic chemical structure of flavones.

Flavonoids are the largest class of plant-specialized metabolites with health-beneficial effects. In plants, they are involved in defense, regulation of metabolism, protection against ultraviolet radiation, and attraction of pollinators [21,22,23]. Flavonoids are characterized by a structure consisting of two benzene rings A and B connected via a heterocyclic ring C (Figure 1) [23,24]. Based on their chemical structure, flavonoids are classified into six subgroups. Among those, the flavones are characterized by a double bond between carbon 2 (C2) and C3 in the C ring and by the attachment of the benzene ring B to C2. Luteolin, chrysin, and apigenin are among the most well-studied flavones. They differ in the number of hydroxyl groups attached to the flavone skeleton. Chrysin has the simplest chemical structure with two hydroxyl groups at C5 and C7 in ring A. Apigenin has an additional hydroxyl group at C4′ in ring B, while luteolin has two additional hydroxyl groups at C3′ and C4′ in ring B (Table 1). Flavones diversity, similarly to other flavonoids, is achieved by chemical modifications and conjugations including hydroxylations, *O*-methylation, or binding to sugars such as *O*- or *C*-glycosides *O*- or *C*-glucuronides, among others. For example, the glycosylation in C8 of apigenin and luteolin produces the flavones vitexin and orientin, respectively. Dimerization of two flavone molecules through different C-C bonds creates a variety of biflavones. For instance, 2′8″-biapigenin is a molecule with powerful anti-inflammatory action commonly found in extracts of *Selaginella tamariscina* [24].

Chrysin is found in fruits such as bitter melon, the wild Himalayan pear, passion fruit, honey, and propolis, and even in some mushrooms [25] (Table 1). Apigenin is abundant in vegetables such as celery, parsley, onions, herbs, and spices such as chamomile, thyme, basil, and oregano. Vitexin and isovitexin are found in abundance in numerous medicinal plants, including hawthorn. Luteolin is present in vegetables such as celery, sweet bell peppers, carrots, green onions, parsley, fennel, broccoli, sage leaves, and chamomile tea. Isoorientin, a *C*-glycoside of luteolin, is found in passion flower and acai palm. Various herbs used in folk and traditional medicine have high levels of flavones. For example, an array of flavones such as apigetrin, baicalein, baicalin, wogonin, scutellarein, and scutellarin are found in the root of Chinese skullcap, *Scutellaria baicalensis*, a plant extensively used in traditional Chinese medicine for the treatment of type 2 diabetes, ulcerative colitis, and respiratory infections [26].

Interestingly, the presence of sugars reduces the absorption of flavones in mammals, with aglycones being absorbed better than glycosylated forms [27,28]. In general, aglycones and mono-glycosides have some limited absorption in the small intestine, while di-glucosides are absorbed primarily in the colon [29]. The average consumption of flavones in humans is estimated to reach several milligrams per day ranging from ~1 mg in Sweden to 9 mg in Italy to 1–3 mg in the U.S.A. and 1–6 mg in China [30,31,32]. Flavones have no adverse effects when consumed as part of the diet. Less clear, however, is the effect of flavone supplements. Commercially available supplements frequently have undetectable amounts of declared ingredients or can be adulterated [33]. Of all the apigenin supplements tested, we demonstrated that only 30% contained apigenin, and those containing apigenin showed lower amounts than reported by the manufacturers, according to our chemical analyses [34]. These findings raise significant concern since supplements are not regulated by the Food and Drug Administration in the U.S.A., despite representing a $35.6 billion industry and being consumed by 70% of the population [35].

When considering the beneficial effects of flavones, their absorption and bioavailability deserve special attention. Flavones are absorbed quickly and excreted in the urine, meaning their bioavailability in humans is relatively low, a characteristic that they share with other flavonoids. For instance, only 0.22% of ingested apigenin could be detected in the daily urine of healthy volunteers [35]. However, other studies detected between 6% and 40% of the ingested apigenin in the urine 12 and 24 h after consumption, respectively [29,36]. Some small amounts of flavones can be absorbed in the upper gastrointestinal tract, with the largest proportion remaining in the gut, where they are further metabolized by the gut microbiota. Emerging studies are currently underway to improve the bioavailability of flavones for human consumption via liposomes, micelles, nanoparticles, or food processing. So far, different flavone nanoparticle formulations evaluated in pre-clinical animal models for their effectiveness and toxicity report encouraging results [37]. For instance, luteolin delivered as nanomicelles inhibited tumor growth by 81% and improved survival without adverse effects on the body weight or histomorphology of vital organs in C57BL/6 mice engrafted with glioma GL261 cells [38]. Novel approaches to increase the bioavailability of flavones via “smart food formulations”, which take into account different food combinations and matrices, are currently underway. For example, food homogenization in combination with heat or chemical processing (cooking, fermentation, and breakdown with digestive enzymes) allows more efficient flavone release and absorption [39]. A mucoadhesive gel containing freeze-dried black raspberries delivered flavonoids into the bloodstream within 5 min after ingestion [40]. Importantly, food preparations with standardized amounts of nutraceuticals may be indispensable for the clinical use of flavones on CID. Epidemiological studies have already established the beneficial effects of flavone consumption on obesity and cancer [41,42,43,44]. However, improving the delivery methods and bioavailability of the flavones will require further investigation.

**Table 1:** Common derivatives of flavones and their source.

Flavone	Hydroxyl Position	Modifications	Source	Ref.
Chrysin	5, 7	-	Bitter melon, wild Himalayan pear, honey, propolis, passion flowers, oyster mushroom	[25]
Apigenin	5, 7, 4′	-	Parsley, celery, onions, chamomile, thyme, basil, oregano, artichoke	[22,29]
Apigetrin	5, 7, 4′	7-*O*-glycoside	Dandelion	[45]
Acacetin	5, 7, 4′	4′- OCH_3_	Black locust, silver birch	[46]
Vitexin	5, 7, 4′	8-*C*-glycoside	Passion flower, chasteberry, bamboo, Hawthorn, fenugreek	[19,47]
Baicalein	5, 6, 7	-	Thyme, *Scutellaria baicalensis*	[26]
Baicalin	5, 6, 7	7-*O*-glycoside	*Scutellaria baicalensis*, *Oroxylum indicum*	[26]
Wogonin	5, 7, 8	8-OCH_3_	*Scutellaria baicalensis*	[26]
Luteolin	5, 7, 3′, 4′	-	Celery, broccoli, bell pepper, parsley, thyme, carrots, rosemary, chamomile, oregano, green onions, fennel, sage	[22,48]
Diosmetin	5, 7, 3′, 4′	4′-OCH_3_	Caucasian vetch, citrus fruit	[48]
Diosmin	5, 7, 3′, 4′	7-*O*-rutinoside, 4′-OCH_3_	Citrus fruit	[48]
Cynaroside	5, 7, 3′, 4′	7-*O*-glycoside	Cumin	[49]
Orientin	5, 7, 3′, 4′	8-*C*-glycoside	Passion flower, buckwheat sprouts, millets	[50]
Isoorientin	5, 7, 3′, 4′	6-*C*-glycoside	Passion flower, Acai palm	[50,51]
Scutellarein	5, 6, 7, 4′	-	*Scutellaria baicalensis*	[26]
Scutellarin	5, 6, 7, 4′	7-*O*-glucurinide	*Scutellaria baicalensis*	[26]
Tangeretin	5, 6, 7, 8, 4′	4′-OCH_3_	Citrus fruit	[48]
Nobiletin	5, 6, 7, 8, 3′, 4′	4′-OCH_3_	Citrus fruit	[48]

## 3. The Innate Immune System and Inflammation

The innate immune system constitutes the first line of defense against pathogens and self-danger stimuli. It is comprised of different cell types. Thus, due to its cellular complexity, this review will focus only on those innate immune cells where the role of flavones has been better studied, including dendritic cells (DC), myeloid-derived suppressor cells (MDSC), monocytes, and macrophages. 

**Figure 2:**
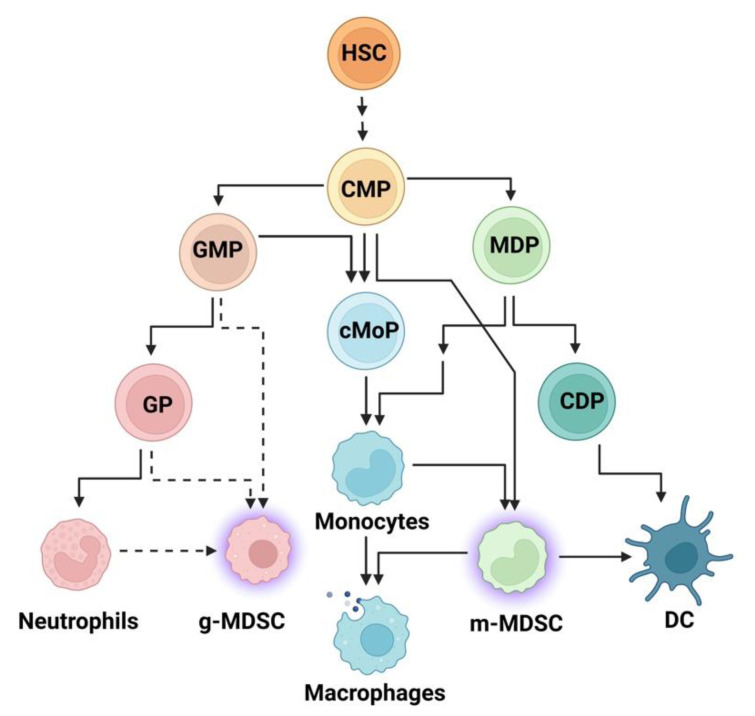
Schematic representation of hematopoiesis. Hematopoietic stem cells (HSC) produce a common myeloid progenitor (CMP) cell, which can give raise to granulocyte-monocyte progenitor (GMP), monocyte-dendritic cell progenitor (MDP), or committed monocyte progenitor (cMoP) cells. GMPs differentiate into granulocyte progenitor (GP), which further differentiates into neutrophils. Dendritic cells (DC) are derived from a common dendritic cell progenitor (CDP). Monocytes can be derived directly from cMoP or from MDP, and they can further differentiate into macrophages or monocytic MDSC (m-MDSC), which can, in turn, switch to DC or macrophages.

Innate immune cells are generated through hematopoiesis in bone marrow (Figure 2). A precursor hematopoietic stem cell (HSC) leads to the formation of a common myeloid progenitor (CMP) cell, which produces granulocyte-monocyte progenitors (GMP) and monocyte-dendritic cell progenitors (MDP). Recent findings using single-cell sequencing show that GMP gives rise to a granulocyte progenitor (GP), a precursor of neutrophils and committed monocyte progenitors (cMoP). MDP can produce either monocytes (through several steps to be further delineated) or dendritic cell progenitors (DCP), which produce DC [52,53,54]. MDSC are classified as granulocyte such as MDSC (g-MDCS) or monocytic (m-MDSC) [55]. The origin of g-MDCS remains poorly understood, and studies suggest that they can derive from GP or GMP [56,57]. M-MDSC can originate from CMP or from monocytes and can differentiate into macrophages or DC [58], highlighting the high plasticity of these cells. 

DC specialize in antigen presentation to the adaptive immune cells. They represent ~1% of mononuclear cells in the blood, but their numbers increase during inflammation. They live from days to weeks and are constantly replenished, a characteristic that they share with monocytes and other immune cells. The number of DC in subcutaneous AT from obese mice and obese individuals is higher than in lean individuals, in numbers that seem to be directly proportional to BMI [59]. The role of DC in obesity and its comorbidities remains poorly characterized. Mice lacking DC, due to the ablation of Flt3l or E2-2 genes, did not develop insulin resistance when fed with a high-fat diet (HFD) [60,61]. However, ablation of the Batf3 gene, which results in the lack of conventional DC, led to the development of insulin resistance and increased adiposity [62]. These controversial findings warrant further studies to delineate the role of specific DC populations in obesity.

In cancer, DC are found in the tumor microenvironment (TME). They regulate anti-tumor immune responses by activating cytotoxic CD8+ and Th1 CD4+ T-cells and by producing a range of cytokines [63]. Studies show that the effectiveness of common anti-tumor therapies such as doxorubicin, epirubicin, and oxaliplatin depends on the recruitment of DC to the TME and the activation of the adaptive immune system, resulting in the induction of immunogenic cell death of cancer cells [11,64,65]. Several strategies are currently being pursued to exploit the anti-tumor properties of DC in cancer immunotherapy [66,67]. However, antibodies and adjuvants utilized to activate DC frequently led to detrimental side effects. From this perspective, the use of natural products to either activate or reduce DC numbers may present a novel approach to controlling immune homeostasis.

Monocytes and macrophages perform similar immune functions including phagocytosis, inflammation, and tissue remodeling. Monocytes comprise ~10% of circulating leukocytes and are short-lived, with the majority of cells undergoing apoptosis after 48 h and a small percentage differentiating into tissue macrophages, which can live longer from months to even years [68,69]. In contrast, macrophages are only found in tissues, and they have a longer lifespan of months. Both monocytes and macrophages comprise heterogeneous populations classified based on the expression of specific protein surface markers [70]. Human monocytes are divided into classical (CD14^+^ CD16^−^, comprising 90% of all monocytes), intermediate (CD14^+^CD16^+^), and non-classical (CD14^−^CD16^+^) monocytes [71]. Recent single-cell RNA-sequencing (scRNA-seq) analyses identified additional molecular heterogeneity in the intermediate monocyte population [72], highlighting the cellular complexity of this lineage. In the mouse, monocytes are classified based on the expression of cell surface markers as classical, expressing Ly6C^hi^CCR2^hi^Cx3Cr1^low^, and non-classical, expressing Ly6C^lo^CCR2^lo^Cx3Cr1^hi^ [70]. Ly6C^hi^ monocytes are counterparts of human classical CD14^+^CD16^−^ monocytes, both sharing high levels of CCR2 expression, a receptor responsible for monocyte migration in response to chemokines such as CCL2, CCL13, CCL8, and CCL7 [73]. Non-classical monocytes from humans and mice express high levels of CX3CR1, a receptor for the CX3CL1 protein responsible for the adhesion of myeloid cells to the blood vessel walls near inflammatory sites [74], indicating the functional evolutionary conservation of this monocyte subset [75]. In inflammatory conditions, human CD14^+^CD16^+^ monocytes leave the bloodstream and differentiate, increasing the pool of tissue macrophages [76]. Classical monocytes produce inflammatory cytokines such as IL-1β, IL-6, IL-8, IL-12, and TNFα and secrete chemokines including monocyte chemoattractant protein-1 (MCP-1 also known as CCL2), CCL7, and CX3CL1 [76]. Obesity is associated with an increased number of classical inflammatory Ly6C^hi^ blood monocytes that positively correlates with fasting insulin levels in the mouse [77]. In obese patients, there was an increased number of classical and intermediate monocytes in the blood that was directly proportional to BMI and waist circumference [78]. Expansion of non-classical monocytes was found in patients with breast and endometrial cancers [79].

Macrophages constitute a heterogenous population with a high degree of cellular plasticity [80], as confirmed by scRNA-seq analyses [81]. The best studied are the M1 and M2 types. M1, or classically activated macrophages, are identified by the expression of CD80, CD86, CD16/32 surface markers and participate in the initiation and progression of the immune response [82]. M2, or non-classically (also named alternatively activated) macrophages, express CD206 and CD163 [82] and contribute to tissue repair and remodeling, angiogenesis, and the resolution of inflammation. Macrophages express numerous receptors including the Toll-like receptors (TLRs), which recognize bacteria, viruses, and saturated fatty acids (FA), among other stimuli [83,84]. Scavenger receptor CD36 facilitates the uptake of long-chain FA. CD36 can also activate TLR 4 and TLR6 to induce inflammatory cytokine production and stimulates the NLRP-3 inflammasome [85]. Signaling via TLR2, TLR4, and CD36 promotes the infiltration of macrophages into obese AT, promoting chronic inflammatory conditions characteristic of obesity [83]. In the TME, VEGF, CCL2, and CD36, among others, facilitate recruitment of monocytes that differentiate into M2 macrophages and stimulate tumor progression and metastasis [86]. M1 macrophages respond to stimuli by producing inflammatory cytokines such as IL-1β, TNFα, IL-6, and IL-12 and chemokines such as MCP-1 and CCL7, among others. M2 macrophages produce anti-inflammatory cytokines such as IL-10 and chemokines including CCL17 and CCL22 [83]. Cellular environmental conditions contribute to M1 and M2 switching. In obesity, for example, macrophage polarization to M1 contributes to the development of metabolic syndrome, due to the production of inflammatory cytokines that exacerbate the development of insulin resistance [83]. Moreover, in the mouse and monkeys in obesogenic conditions, there is a 1:1 ratio of M1 to M2 macrophages, contrasting with a 1:4 M1 to M2 ratio found in lean animals [87,88]. These findings demonstrate that with obesity, there is an increased number of M1 macrophages, which promote chronic inflammatory conditions.

Cancer cells produce IL-10, CCL2, and VEGF that induce M2 macrophage polarization, promoting tumor immune evasion [89]. Mass single-cell cytometry analyses of 144 human breast cancer and 46 adjacent non-tumorous tissues revealed that the tumor promoted the accumulation of M2 tumor-associated macrophages (TAM) [90]. An increased number of TAM is associated with poor prognosis in cancers, including ovarian, breast, colorectal, bladder, and lung cancer [91,92,93,94]. Additionally, TAM promote tumor resistance to chemotherapy [95,96,97]. Conversely, ablation of TAM with the drug legumain reduced tumor growth and metastases in a murine model of breast cancer [98]. Therapies to decrease TAM, decrease their recruitment to the tumor, or reprogram TAM from M2 to M1 are currently underway in clinical trials [99,100].

MDSC are a heterogeneous population, comprised of granulocytic (g-MDSC) or monocytic (m-MDSC) like types. MDSC are found usually in circulation and their numbers increased in blood and tissues with CID. Obese subjects have a higher number of m-MDSC in the blood than lean controls, and that number positively correlates with their BMI [78]. In obesity, MDSC-induced production of IL-10 contributes to M2 polarization of macrophages and may be beneficial to counteract inflammation [101]. The levels of g-MDSC increase in the blood of patients with colorectal cancer, with the highest number observed during stage IV of the disease [102]. Additionally, the total amount of both m- and g-MDSC positively correlates with the number of organs affected by metastases [102]. In the TME, m-MDSC can differentiate into macrophages or DC [103,104]. The presence of MDSC in the TME promotes tumor growth and resistance to therapy. The number of m-MDSC has been negatively correlated with chemotherapy resistance in breast, prostate, lung, and colorectal cancers [57,105,106,107]. Overall, myeloid cells are good candidates for targeted cell therapies in CID. Natural flavones that modulate the number and type of immune cells may have great potential as preventive or therapeutic strategies for CID. 

## 4. Inflammatory Pathways

Inflammation is orchestrated by a network of central transcription factors (TF) that regulate the expression of inflammatory regulators. Here, we briefly summarize the key signaling pathways contributing to inflammation which are impacted by flavones.

### 4.1. NF-κB

**Figure 3:**
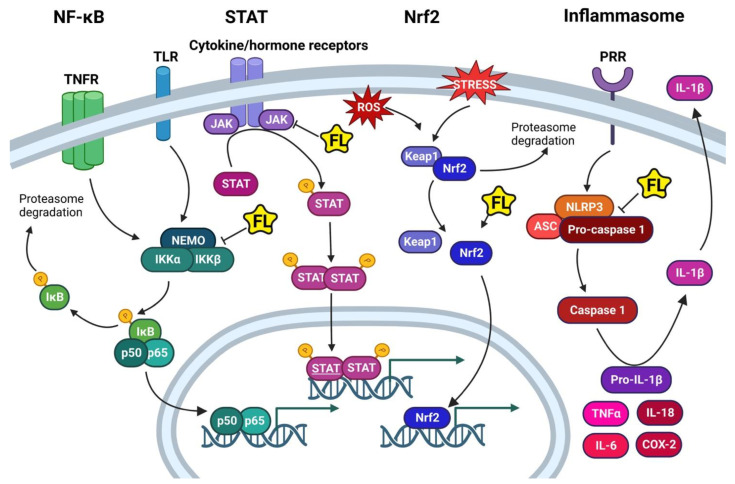
Major intracellular signaling pathway involved in control of CID. The NK-κB pathway is activated by inflammatory stimuli acting through tumor necrosis factor receptors (TNFR) and Toll-like receptors (TLR). The signal transducer and activator of transcription (STAT) pathway is activated by hormones and cytokines. The (NF-E2-related factor 2) Nrf2 signaling pathway is a major sensor of reactive oxygen species (ROS) and oxidative stress in the cell. The inflammasome pathway is activated by stimuli from pattern recognition receptors (PRR) or by extracellular signals such as pathogens, crystals, and cholesterol. Flavones (FL) inhibit NF-κB signaling via binding to the kinase complex IKKα, IKKβb, and NEMO. Flavones inhibit STAT signaling via binding to JAKs. Flavones stimulate the transcription of ARE-dependent genes by Nrf2. Flavones decrease expression of inflammasome genes NLRP3 and caspase-1.

Nuclear factor kappa-light-chain-enhancer of activated B cells (NF-κB) represents a family of TF that regulates the expression of genes involved in the regulation of the immune response [108]. The family includes five structurally related proteins including RelA (p65), RelB, c-Rel, NF-κB1 (p105/p50), and NF-κB2 (p100/p52), which have a common Rel Homology Domain (RHD) necessary for protein dimerization and DNA binding. NF-κB1 and two are produced by proteolysis of p105 and p100 to yield p50 and p52, respectively. The RHD domain of NF-κB proteins binds to the inhibitory protein nuclear factor of kappa light polypeptide gene enhancer in B-cells inhibitors (IκB). The IκB family, comprised of IκBα, IκBβ, and IκBε, is characterized by the presence of ankyrin repeats responsible for their association with NF-κB [108]. NF-κB is usually found in the cytoplasm forming dimers, which are kept inactive through the association with IκB inhibitory proteins (Figure 3). Phosphorylation of IκB by the IκB kinases (IKK) promotes IκB rapid proteolytic degradation, freeing NF-κB and allowing relocalization into the nucleus to activate transcription. IKK is comprised of two catalytic kinases IKKα, IKKβ, and NEMO, a regulatory subunit [109]. IKK activation can be triggered by several kinases such as TGFβ-activated kinase (TAK), which can also activate MAP kinases p38 and JNK or MKK [109]. 

The activation of NF-κB leads to the expression of genes involved in inflammation, proliferation, cell survival, and apoptosis. The NF-κB pathway is crucial in obesity and it has been associated with hyperglycemia, hyperlipidemia, insulin resistance, and hepatic steatosis (reviewed in [110]). Recent RNA-seq studies of human adipocytes from lean and obese patients revealed an enhanced NF-κB gene signature and increased activation of the NF-κB pathway in white adipose tissue (WAT) of obese patients, especially of those with metabolic syndrome and type 2 diabetes [111,112]. Likewise, in the mouse, obesity caused by the consumption of a Western diet showed activated NF-κB signaling [112]. Conversely, inhibition of NF-κB in obese animals by a selective NF-κB inhibitor, celastrol, ameliorates pathophysiological processes such as obesity-induced kidney injury, insulin resistance, and hepatic steatosis [113,114,115]. 

The NF-κB pathway also links chronic inflammation and tumor development. Activation of NF-κB in the TME leads to increased proliferation of tumor cells due to the activation of cyclin D1 and c-Myc [115,116]. Additionally, NF-κB triggers the avoidance of programmed cell death due to the stimulation of anti-apoptotic genes, enhances angiogenesis due to the production of VEGF, and increases metastasis due to the expression of matrix metalloproteinases and cell adhesion molecules [109,117]. The signaling via the p65 NF-κB subunit seems to be critical for macrophage polarization towards the anti-tumor M1 phenotype [118]. Overall, activation of the NF-κB pathway contributes virtually to all hallmarks of cancer and may provide a novel target for cutting-edge therapies [119,120,121]. Therefore, understanding how to control the NF-κB pathway may present unique opportunities for reducing immune dysregulation and controlling cancer development and growth. 

### 4.2. STAT Pathway

The signal transducer and activator of transcription (STAT) pathway is involved in regulation of the immune response, proliferation, differentiation, and apoptosis. Dysregulation of the STAT signaling pathway contributes to CID such as obesity and cancer. The STAT family includes STAT1, STAT2, STAT3, STAT4, STAT5a, STAT5b, and STAT6 proteins [122]. The binding of immune cytokines such as IL-6, IL-4, or hormones such as leptin to cognate receptors triggers the dimerization of the receptors, which, in turn, induces the activation of Janus kinases (JAK). There are four different JAK kinases including JAK1, JAK2, JAK3, and Tyk2 [122]. JAK phosphorylates cytoplasmic STAT, inducing its dimerization and translocation to the nucleus to regulate the expression of genes involved in inflammation (Figure 3). 

The STAT pathway is triggered by cytokines inducing a signaling cascade during inflammation. IFN-γ stimulation of STAT1 induces the production of inflammatory cytokines including TNFα and IL-1β in macrophages [122]. IL-4 and IL-13 stimulate STAT6 which induces M2 polarization of macrophages and enhances the expression of arginase 1 (Arg1). In the TME, STAT3 signaling promoted the M2 polarization of TAM, enhanced IL-10 expression, and facilitated an immunosuppressive environment [123]. 

The contribution of the STAT pathway to obesity was clearly revealed by studies in mice with genetic ablations of key molecules in the pathway. Deficiencies of JAK3 led to enhanced insulin resistance, increased adipose weight, and hepatic steatosis in mice fed with HFD [124]. In contrast, genetic ablation of STAT4 resulted in reduced inflammation in AT and improved insulin sensitivity in HFD-induced obesity [125]. Because persistent activation of the STAT pathway is involved in the pathogenesis of several CID, multiple chemical inhibitors of JAK are currently being tested as therapeutic interventions with the second generation of such chemicals currently in the pipeline [126]. However, adverse effects of JAK inhibitors such as blood clots and liver damage prompt the need to utilize natural substances with minimal side effects for control of the STAT pathway in inflammation, obesity, and cancer.

### 4.3. Nrf2 Pathway

Nuclear factor erythroid 2-related factor 2 (Nrf2) is a basic leucine zipper transcription factor, acting as a central protector against biotic and abiotic stress factors. Nrf2 is ubiquitously expressed, regulates inflammatory states, and has a key protective role against oxidative and metabolic stress [127]. Under normal physiological conditions, Nrf2 undergoes ubiquitination and proteasome degradation due to its association with a dimer of the inhibitory protein Kelch-like ECH-associated protein 1 (Keap1) (Figure 3). Under stress conditions, Keap1 inhibits the ubiquitination of Nrf2 and releases it, allowing its nuclear translocation. In the nucleus, Nrf2 binds to antioxidant response elements (ARE) to activate the transcription of ARE-dependent genes, including NADH dehydrogenase (NQO1), glutathione synthase (GSH), superoxide dismutase (SOD), heme oxygenase (HO-1), and catalase (CAT). These genes, in turn, decrease levels of free radicals and oxidative stress in the cell. The Nrf2 pathway interacts with NF-κB signaling to regulate the cellular redox balance during inflammatory states. Nrf2 functionally inhibits the NF-κB pathway by several mechanisms. It creates a reducing environment in the cell, counteracting the release of ROS induced by NF-κB [128]. Nrf2 inhibits the proteasomal degradation of IκBα and thus, prevents the nuclear translocation of NF-κB [129]. Nrf2 also suppresses transcription of NF-κB-dependent proinflammatory genes TNFα, IL-6, and IL-1β [128,130]. 

The activation of the Nrf2 pathway has been linked to improved outcomes in diabetes and cancer [131,132]. Mice with genetic ablation of Keap1 or Nrf2 in the whole body are partially protected against HFD-induced obesity [131,132]. However, the effects of Nrf2 on obesity may be tissue specific. For instance, the ablation of Nrf2 in adipocytes leads to enhanced insulin resistance, while Nrf2 deletion in hepatocytes results in increased insulin sensitivity [133]. Overall, accumulating evidence supports the importance of the Nrf2 pathway in obesity. In cancer, Nrf2 plays contradictory roles depending on the cellular environment [127]. This pathway is critical for chemoprevention and tumor suppression. However, in already established tumors, Nrf2 may promote tumor progression by protecting it from oxidative stress and inducing angiogenesis. Indeed, genetic ablation of Nrf2 in mice is associated with increased tumor burden after carcinogen exposure, supporting its role in tumor prevention [134,135]. At the same time, Nrf2 is overexpressed in different tumor types [136,137] and can promote tumor growth and metastasis [138]. Furthermore, Nrf2 promoted cancer chemoresistance by stimulating the expression of ARE-dependent multidrug resistance genes and decreased the effectiveness of common chemotherapies such as doxorubicin, carboplatin, or cisplatin [139,140]. Importantly, Nrf2 is a central regulator of MDSC number and function. Nrf2 activation is essential for the maintenance of MDSC in undifferentiated states and is necessary for MDSC-mediated immunosuppression [141]. Given the importance of the Nrf2 pathway in health and disease, there are multiple studies underway to exploit Nrf2 modulation for therapeutic purposes.

### 4.4. The Inflammasome Pathway

The (NOD-, LRP- and pyrin domain-containing protein 3) NLRP3 inflammasome pathway is an essential mediator of the innate immune system that detects microbial toxins and microenvironmental stimulants, such as uric acid crystals, cholesterol, and asbestos, among others [142]. NLRP3 inflammasome involves the formation of a multiprotein complex containing pro-caspase-1 that, upon activation, regulates the release of proinflammatory cytokines IL-1β and IL-18 (Figure 3). Activation of the NLRP3 inflammasome pathway requires two events. The first signal is the activation of NF-κB and synthesis of pro-IL-1β. The second event is an assembly of the inflammasome complex that involves the recruitment of inactive pro-caspase-1 via the linker protein ASC [142]. This leads to autocatalytic activation of pro-caspase-1 into active caspase-1, which cleaves pro-IL-1β and pro-IL-18 producing immunologically active IL-1β and IL-18. Released cytokines, in turn, stimulate inflammatory cell death and enhance inflammatory processes. While activation of the NF-κB pathway is a prerequisite for NLRP3 inflammasome formation, Nrf2 has an opposing effect and inhibits ROS production, which is stimulatory for the NLRP3 inflammasome. Additionally, Nrf2 reduces the expression of genes involved in inflammasome assembly, such as caspase-1, NLRP3, IL1β, and IL-18 [143].

Inappropriate NLRP3 inflammasome activation is implicated in the pathogenesis of a variety of CID including obesity and cancer [142]. For instance, the NLRP3 inflammasome senses danger signals from HFD and promotes low-grade inflammation and insulin resistance in AT [144]. Accordingly, mice deficient in NLRP3 do not develop obesity and insulin resistance while fed with HFD [145]. Inflammasome components, including NLRP3, are expressed at higher levels in the AT of obese patients compared to lean patients [146]. In some cancers, the NLRP3 inflammasome may have a protective role. For instance, mice lacking NLRP3 exhibited increased metastases of colorectal cancer [147]. Furthermore, injections of IL-18 into NLRP3-deficient mice to compensate for the inflammasome’s absence led to a lower number of primary tumors [147]. Conversely, in a mouse model of orthotopic breast cancer, the lack of either NLRP3 or caspase-1 genes resulted in fewer lung metastases, suggesting that the NLRP3 inflammasome is promoting cancer [148]. These tumor-promoting effects were mediated by the production of IL-1β and the recruitment of immunosuppressive MDSC into the tumor [148]. Importantly, NLRP3 inflammasome activation may counteract the efficacy of chemotherapeutic agents such as 5-fluorouracil or gemcitabine [149]. Altogether, the NLRP3 inflammasome may play contrasting roles in metabolic diseases and tumorigenesis, thus creating multiple directions for future studies of its function in diverse pathological states.

## 5. Chronic Inflammatory Diseases: Obesity and Cancer

Chronic inflammation is a common feature of numerous pathological conditions. Uncontrolled inflammation, oxidative stress, tissue damage, and dysregulation of the innate immune system cell repertoire are frequently observed in patients with obesity and cancer. Furthermore, obesity itself is considered an additional risk factor for a variety of cancer types. It is anticipated that cancer incidence will continue to surge in the future due to the increased prevalence of sedentary lifestyles, metabolic syndrome, obesity, and inflammation. 

### 5.1. Obesity-Induced Inflammation

**Figure 4:**
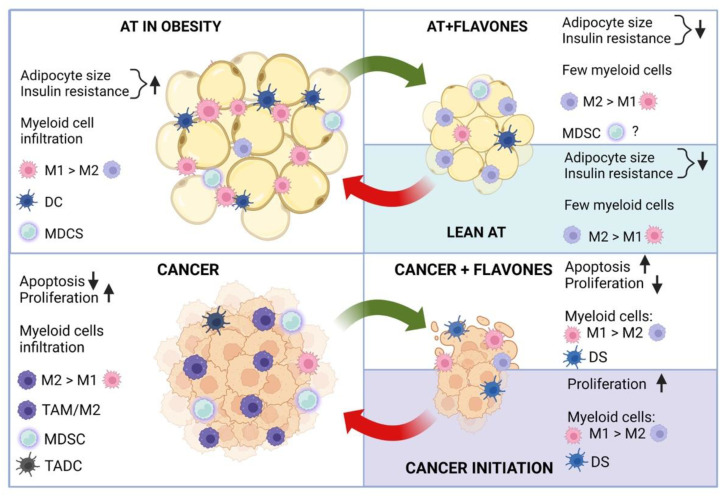
The effect of flavones on the innate immune cells in obesity and cancer. In obesity, flavones exhibit anti-inflammatory and anti-obesogenic action in adipose tissue (AT) by decreasing infiltration of dendritic cells (DC) and switching macrophage polarization to the M2 phenotype. The effect of flavones on myeloid-derived suppressor cells (MDSC) in obesity is not known. In cancer, flavones decrease infiltration of tumor-associated macrophages (TAM), reduce the number and function of MDSC, activate functionally deficient tumor-associated DC (TADC), and promote macrophage polarization toward the anti-tumor M1 phenotype. ↓—decrease, ↑—increase, ?—unknown.

According to the World Health Organization, people with a body mass index (BMI) of 25–29.9 kg/m^2^ are considered overweight and people with a BMI ≥ 30 kg/m^2^ are considered obese. Obesity causes increased morbidity and mortality and presents an economic burden around the globe. Obesity is currently recognized as a CID. Adipose tissue (AT), particularly visceral fat, is a metabolically active organ that stores energy in the form of lipids and produces hormones and cytokines (collectively called adipokines) and a variety of inflammatory molecules. The hormone leptin produced by “obese” adipocytes stimulates STAT signaling and promotes inflammation, whereas the hormone adiponectin, normally produced in “lean” AT, is generally anti-inflammatory. In healthy lean individuals, adipocytes produce predominantly anti-inflammatory cytokines including IL-4, IL-10, IL-13, and TGFβ that promote a predominance of M2 macrophages (Figure 4). The intake of HFD induces larger adipocyte size and metabolic changes that alter the production of inflammatory cytokines, including TNFα and IL-1β, and chemokines such as MCP-1 and CCL7. These changes in the AT environment increase the recruitment of monocytes into the AT and their differentiation into M1, inflammatory macrophages. Furthermore, AT in obese or overweight people create inflammatory conditions systemically, with increased levels of IL-6, IL-8, TNFα inflammatory cytokines, and VEGF [9]. Overall, obesity results in low-grade chronic inflammation which contributes to the development of metabolic syndrome [150].

The infiltration of innate immune cells into AT is a key feature associated with obesity in both humans and mice [9,83]. MCP-1 is a critical chemokine necessary for the infiltration of monocyte and macrophages into obese AT [150]. The number of infiltrating monocytes/macrophages and levels of expression of proinflammatory cytokines is directly proportional to the magnitude of insulin resistance and metabolic dysregulation in the adipocytes. Moreover, NLRP3-induced IL-1β production promotes the development of insulin resistance [145]. Consistently, genetic silencing of TNFα in AT macrophages was sufficient to improve insulin sensitivity systemically [151]. 

With the onset of obesity, DC are attracted to the AT through CCR7 signaling [150,152]. The number of DC is comparable with the number of macrophages in obese AT and they substantially contribute to the development of insulin resistance independently from monocytes and macrophages [150]. Conversely, a deficiency of DC protects against HFD-induced metabolic disorders [13,153]. 

The role of MDSC in obesity is not fully elucidated. An increased number of m-MDSC was detected in the blood of obese individuals and diabetic patients [154,155]. Analogously, HFD-induced obesity increased the numbers of MDSC in the murine WAT. The ablation of MDSC in obese mice enhanced insulin resistance and exaggerated systemic inflammation, whereas the transfer of purified MDSC improved glucose tolerance and insulin sensitivity in obese mice [101]. It is proposed that MDSC provide a checks-and-balances system in obese tissues. Given the immunosuppressive nature of MDSC, their accumulation in obese patients systemically may facilitate tumorigenesis and provide at least one mechanism by which obesity exacerbates cancer. 

Numerous epidemiological studies showed the relationship between excessive weight and cancer. Women with breast cancer who had a greater BMI had significantly worse disease-free survival than women with a lower BMI [156]. Central adiposity or fat mass around the abdomen seems to be the most detrimental to human health. Unfortunately, not many studies have assayed a correlation between abdominal obesity (visceral fat) and cancer risk. In particular, a higher waist circumference and waist-to-hip ratio (an indicator of visceral fat deposits) are associated with an increased risk of lung cancer [157] and increased mortality from pancreatic cancer [158], regardless of BMI. Women who were obese at the time of breast cancer diagnosis had increased cancer-associated mortality [159,160]. Furthermore, a weight gain of 10% or more after breast cancer diagnosis and chemotherapy increases overall mortality by 23% [161]. Conversely, weight loss is associated with decreased risk of cancer and improved disease-free survival in patients with breast cancer [162].

### 5.2. Cancer-Induced Dysregulation of Innate Immunity

Inflammation contributes to all stages of cancer development: initiation, promotion, growth, and metastasis [163,164]. During the tumor-initiating phase, DC activate the adaptive immune response and drive M1 macrophage polarization (Figure 4). Inflammatory cytokines such as IL-1β, TNFα, and IFNγ are produced to combat the initial tumor via activation of NF-κB, STAT1, and NLRP3 inflammasome pathways. However, as tumors develop, a persistent inflammatory environment stimulates ROS production contributing to DNA damage in tumor cells, driving malignant transformation, and switching the innate immune repertoire. MCP-1 expression in the tumor promotes the recruitment of monocytes and MDSC [165]. In the TME, infiltrating classical monocytes differentiate into TAM and promote tumor development, while non-classical monocytes promote angiogenesis [166]. Several studies have shown an association between a higher number of macrophages in the TME and poor cancer prognosis [167,168]. Accordingly, the chemical depletion of TAM reduced tumor growth in several experimental cancer models [169,170]. Tumor cells release transforming growth factor-beta 1 (TGF-β1), IL-4, IL-6, and IL-10 that educate TAM to polarize into M2 or become immunosuppressive [99,167,171]. The overexpression of the p50 NF-κB subunit, which lacks a transcription transactivation domain, in TAM prevents M1 macrophage activation but does not influence the production of anti-inflammatory IL-10 [172]. IL-10 and VEGF inhibit the activation of DC, reducing the activation of the adaptive immune system. Selective inhibition of VEGF increased the number of DC in human triple-negative breast cancer MDA-MB-231 xenografts [173]. In addition, impaired DC function was reversed by antibodies against IL-10 combined with immunostimulatory oligonucleotide CpG [174]. During tumor development the number of MDSC in the TME increases, where they suppress anti-tumor cytotoxic responses via activation of STAT3 [175,176]. Consequently, the inhibition of STAT3 with a chemical inhibitor JSI-24 significantly delayed mammary tumor growth in mice engrafted with 4T1 cells [176]. Remarkably, a greater number of MDSC accumulated in the blood and TME in obese mice with mammary carcinoma compared to lean ones [177], highlighting the importance of obesity in the dysregulation of innate immune cells. Furthermore, MDSC isolated from obese animals were more immunosuppressive than MDSC from lean mice [177]. Altogether, as the tumor progresses, it creates an immunosuppressive environment conducive to tumor growth. 

Given that cancer development and progression are tightly regulated by innate immune cells and inflammation, anti-inflammatory drugs are proposed as a supplementary treatment for cancer prevention and therapy. Multiple NSAIDs and steroids are used daily to curb excessive inflammation. Problematically, these pharmaceuticals usually impart severe side effects on other organs and systems. Therefore, more attention needs to be drawn to natural compounds with powerful anti-inflammatory properties. 

## 6. Anti-Inflammatory Mechanisms of Flavones

The beneficial effects of flavones on obesity and cancer are well documented and have been attributed in great part to their ability to reduce inflammation (Table 2) [43,44]. Flavones exhibit anti-obesogenic effects, improve glucose homeostasis, and reduce metabolic syndrome [178,179,180,181]. In epidemiological studies, a plant-based Mediterranean diet (MD) enriched in flavones apigenin and luteolin provides multiple health benefits, including decreased incidence of diabetes, reduction in waist circumference, and greater weight loss in intervention studies in obese patients [182,183,184]. Multiple preclinical studies showed that chamomile tea, which contains large quantities of apigenin and luteolin, (up to 1.2% of apigenin), decreases metabolic syndrome and improves insulin sensitivity [185,186,187,188]. Remarkably, greater adherence to MD also decreases the risk of several cancers, particularly, triple-negative breast cancers [189,190]. A population-based study has demonstrated that the highest intake of foods enriched in apigenin is negatively associated with ovarian cancer risk [191]. Furthermore, the consumption of a diet recommended by the American Cancer Society enriched in fruits and vegetables improves survival in patients with colorectal cancer by 42% [192]. We showed that the celery-based apigenin-rich diet (CEBAR, providing 50 mg/kg/day apigenin) increases apigenin bioavailability [27]. CEBAR shows potent systemic anti-inflammatory activity reducing the levels of TNF and inflammatory microRNAs [193]. These results demonstrate that medically active concentrations of apigenin can be delivered by formulated diets rich in this flavone. Evidently, future studies of whole foods with standardized amounts of the most abundant flavones will delineate their role in chemoprevention and cancer intervention. Current epidemiological studies and results from cellular and model systems have demonstrated the ability of flavones to change the repertoires of innate immune cells and affect the cytokine and chemokine production that define the immune characteristics of the TME.

### 6.1. Flavones and Innate Immune Cell Repertoires in Obesity

Studies in animals with HFD-induced obesity demonstrated that flavones markedly reduce inflammation and metabolic syndrome in obese mice—they decrease triglycerides, cholesterol, and blood glucose levels; prevent liver injury, and improve insulin sensitivity (Table 2). Flavones are potent regulators of innate immune cells in obesity. Within a large range of doses and via different routes of administration, flavones uniformly decrease the infiltration of innate immune cells to obese AT (Figure 4). Apigenin, tangeretin, and luteolin attenuated obesity-induced inflammation by significantly decreasing the infiltration of macrophages in AT [194,203,205]. In addition, chrysin decreased monocyte recruitment to AT and the differentiation of monocytes to macrophage, luteolin significantly reduced the infiltration of CD11c + DC, while baicalin decreased the proportion of classical proinflammatory CD11b + Ly6C^hi^ monocytes in the blood [200,202,203]. Moreover, flavones shifted polarization of AT macrophages from proinflammatory M1 to the anti-inflammatory M2 phenotype [194]. In particular, chrysin reduced the expression of M1 markers such as CD80, CCR7, and CCL3; while both chrysin and tangeretin increased the expression of M2 marker genes such as CD206, Arg1, and Ym1 [202,205].

At the molecular levels, apigenin treatment in vivo inhibited the NF-κB pathway via the activation of PPARγ, a nuclear ligand-activated transcription factor, in macrophages [217]. Importantly, when compared with anti-diabetic drugs thiazolidinediones, apigenin is as potent in reversing inflammation and metabolic disorder. However, it lacks the adverse effects commonly associated with PPARγ agonists, which include cardiovascular failure, liver toxicity, bone fractures, and potential carcinogenesis [194]. Similarly to apigenin, chrysin also exhibits weak agonistic activity for PPARγ in macrophages [218]. The activation of PPARγ by chrysin mediates a switch from M1 to M2 polarization in AT macrophages [202]. Apigenin binding to PPARγ decreased NF-κB-dependent production of inflammatory cytokines TNFα, IL-6, IL-1β, and chemoattractant MCP-1 [194]. In addition, apigenin (10 μM) reduced cytokine release from monocytes by inhibiting the activity of IKK and decreasing phosphorylation of NF-κB p65 in vitro [219] and by reducing the activity of NF-κB in vivo [220]. Chrysin, tangeretin, and luteolin also reduced levels of NF-κB-dependent proinflammatory cytokines TNFα, IL-6, and IL-1β in the blood and AT, while also decreasing the expression of chemoattractant MCP-1 [123,180,202,203,205]. Interestingly, luteolin treatment also decreased the expression of another monocyte chemoattractant CCL7, and genes involved in NF-κB signaling such as TLR4, CD14, and IRF5 [203]. 

Luteolin (0.005% *w*/*w* in HFD) decreased the steady-state mRNA expression and protein levels of some of the major NLRP3 inflammasome components, including Nlrp3, Asc, and caspase-1, in the gonadal AT from ovariectomized “postmenopausal” mice [215]. In addition, luteolin decreased NLRP3-dependent secretion of IL-1β from murine macrophages in vitro in a dose-dependent manner, inhibited caspase-1 activation, and prevented ASC oligomerization [215]. Remarkably, these effects of luteolin are comparable to MCC950, a selective inhibitor of the NLRP3 inflammasome, but without the adverse effects of MCC950 such as renal glomerulosclerosis [221,222].

Numerous studies have demonstrated molecular mechanisms underlying the anti-inflammatory action of flavones in a co-culture of adipocytes and immune cells (Table 2). For example, nobiletin reduced the elevated secretion of NF-κB-dependent TNFα and MCP-1 from macrophage cells RAW264.7 co-cultured with differentiated adipocytes 3T3-L1 in a dose-dependent manner [204]. Simultaneously, nobiletin increased the expression of an Nrf2-dependent gene HO-1 in both cell types. Silencing of HO-1 partially blocked the anti-inflammatory effect of nobiletin [204]. Interestingly, nobiletin is also a ligand for the nuclear hormone receptor RORα [223], important for macrophage polarization toward the M2 phenotype [224]. Acacetin decreased the expression of NF-κB-dependent genes MCP-1, IL-6, and TNFα in macrophages stimulated by culture media obtained from differentiated adipocytes [199]. Acacetin, in a range of doses from 3 to 100 μM, inhibited phosphorylation of IκBα and prevented nuclear translocation of the p65 NF-κB [199]. Likewise, tangeretin modulated a cross-talk between bone marrow-derived macrophages and adipocytes. It decreased the expression of M1 markers IL-6, IL-1β, TNFα, and MCP-1, while increasing the expression of M2 markers Arg1, CD206, and IL-10 in AT [205]. Altogether, in addition to decreasing adiposity, flavones inhibit infiltration of DCs and monocytes in AT, decrease the M1/M2 ratio, and reduce the expression of proinflammatory cytokines and chemokines. Since cytokines contribute to insulin resistance, flavone action improves insulin sensitivity in AT (Figure 4). Currently, it remains to be established how flavones regulate the function of MDSC in obese AT. Based on existing data, one can hypothesize that flavones enhance the function of MDSC recruited to obese AT. 

### 6.2. Flavones Control Innate Immune Cell Repertoires and Inflammation in Cancer

The anti-cancer effects of flavones are well documented [225,226]. In addition to direct anti-proliferative and pro-apoptotic actions, flavones modulate immune surveillance by the innate immune cells and control tumor-associated inflammation. When given in vivo, apigenin (30 mg/kg body weight *i.p*. injected) and luteolin (30 mg/kg body weight *i.p*. injected) treatments reduced the size of non-small cell lung carcinoma (NSCLC) H358 and Lewis lung carcinoma xenografts in mice [208]. Apigenin treatment produced stronger activation of anti-tumor CD8+ cytotoxic cells and greater production of anti-tumor cytokines IFNγ, TNFα, and granzyme B in the blood than luteolin [208]. In another study, apigenin (150 mg/kg/day administered via oral gavage) significantly reduced tumor size, increased the abundance of CD4+ and CD8+ cytotoxic cells, and decreased the expression of immunosuppressive protein PD-L1 on DC isolated from mice xenografted with melanoma B16-F10 cells [207], suggesting that apigenin treatment improved the functional activity of DCs. In a similar fashion, apigenin (30 μM) inhibited the expression of PD-L1 in human DCs and stimulated the greater cytotoxic activity of DC against human A375 melanoma cell lines [207].

Flavones exhibit anti-tumor effects by regulating the number and function of MDSC. For instance, chrysin (20 and 40 mg/kg/day *i.p.* injected) significantly decreased tumor volume in mice with murine melanoma B16-F10 xenografts and decreased the number of g-MDSC in the bone marrow and spleens [227]. Chrysin inhibited the production of NO and ROS specifically in g-MDSC but not in m-MDSC. Moreover, chrysin decreased Arg1 and COX-2 steady-state mRNA expression, reduced MDSC proliferation, and increased the proportion of cytotoxic CD8+ cells [227]. Similarly, apigenin (25 mg/kg/day *i.p.* injected three times per week) reduced the number of g-MDSC and immunosuppressive TAM and increased mobilization of anti-tumor CD8^+^ cells in mice with orthotopic pancreatic cancer [195].

Flavones alter the polarization of TAM in the TME. Baicalein (50 mg/kg *i.p.* injected every other day) significantly delayed tumor growth and increased the percentage of M1 anti-tumor macrophages in both mouse xenografts of melanoma B16-F10 cells and murine mammary cancer 4T1 cells [201]. Likewise, vitexin (40 and 160 mg/kg/day *i.p.* injected) promoted M1 polarization in colitis-associated colon cancers but simultaneously induced M2 macrophage polarization (as judged by a number of CD206^+^ macrophages) in noncancerous tissue adjacent to the tumor [206]. Additionally, vitexin decreased the expression of proinflammatory cytokines TNF-α, IL-1β, and IL-6 and elevated levels of anti-inflammatory IL-10 to curb inflammation and support tissue repair in non-cancerous tissue (Table 1). This is particularly interesting since vitexin acts as an anti-inflammatory factor in normal tissue and as an anti-tumorigenic factor in cancers. Such a dichotomous effect of flavones on macrophages in cancer versus normal tissue is not fully understood and warrants further investigation. 

The beneficial effects of flavones in cancer may be, to a larger extent, attributed to the inhibition of the major inflammatory pathways. Apigenin (200 and 300 mg/kg/day given via oral gavage) delayed the development of hepatocellular carcinoma (HCC) in nude mice xenografted with PLC/PRF/5 cells and improved survival in a dose-dependent manner [196]. The higher apigenin dose (300 mg/kg/day) significantly decreased levels of nuclear NF-κB protein in HCC tumors [196]. Apigenin in doses equivalent to human consumption in a healthy diet (20–50 μg/day via oral gavage) significantly reduced the growth of prostate cancers in mice, inhibited NF-κB p50 and p65 phosphorylation, and decreased phosphorylation and degradation of IκBα in a dose-dependent manner [197]. Interestingly, baicalein increased TNFα expression in M1 macrophages isolated from murine tumors via NF-κB pathway, phosphorylation of IκB, and nuclear translocation of p65 [201].

Molecular docking studies showed that acacetin exhibited a strong binding with JAK2, results that suggested its promising role as a JAK2 inhibitor, while baicalin showed strong interaction with STAT1 and STAT4 [228]. Luteolin inhibited JAK1-dependent STAT1 phosphorylation, resulting in reduced IL-8 and COX-2 production in HT-29 colon cancer cells [209]. Similarly, luteolin, in a dose-dependent manner, decreased IL-6-induced STAT3 phosphorylation in cholangiocarcinoma cells KKU-M156 [210]. Both apigenin and luteolin (from 10 to 50 μM) inhibited STAT1 and STAT3 phosphorylation and decreased expression of STAT-dependent programmed death-ligand 1 (PD-L1), a major factor responsible for the suppression of the adaptive anti-tumor immune response in NSCLC and melanoma cell lines [207,208].

The Nrf2 pathway is also modulated by flavones. Tangeretin (40 and 60 μM) inhibited Nrf2 levels in drug-resistant A549 lung cancer cells, induced apoptosis, and reduced tumor growth in combination with the chemotherapeutic drug paclitaxel in vivo in A549 xenografts [213]. Similarly, wogonin (20–60 μM) inhibited the growth of breast cancer MCF-7 cells resistant to doxorubicin in a dose-dependent manner by decreasing Nrf2 protein levels in the nucleus and reducing the production of Nrf2-dependent gene HO-1 [214]. While inhibition of Nrf2 is beneficial in drug-resistant cancers, Nrf2 activation may be important for cancer chemoprevention. From this perspective, flavones deserve more attention. Indeed, apigenin and luteolin (1.5–6.25 μM) activated the ARE-luciferase reporters in liver hepatocellular carcinoma HepG2-C8 cells, indicating that flavones directly stimulated Nrf2-dependent transcription [211].

Activation of the NLRP3 inflammasome signaling has the potential to inhibit cancer growth. Molecular docking analyses identified that luteolin strongly interacts with NLRP3 inflammasome [216]. Luteolin (50–150 μM) increased the expression of NLRP3 protein, induced activated caspase-1, and stimulated the production of IL-1β in colorectal HT-29 cells in vitro. In addition, luteolin treatment (50 mg/kg/day *i.p*. injected) increased ASC, cleaved caspase-1, IL-1β, and NLRP3 protein levels in xenografts of HT-29 cells in nude mice, simultaneously decreasing tumor size [216]. 

Collectively, flavones exhibit potent anti-tumorigenic action via control of the innate immune cells in the TME (Figure 4). Flavones stimulate the functional activity of DC, inhibit the immunosuppressive function of MDSC, and shift TAM polarization from tumor-promoting M2 toward the anti-tumor M1 phenotype. Combined with potent pro-apoptotic and anti-proliferative effects, flavone action leads to tumor apoptosis and regression. 

## 7. Conclusions and Future Remarks

The anti-inflammatory activity of flavones derived from cellular and in vivo animal models is indisputable. Flavones effectively regulate major inflammatory pathways, averting the development of metabolic disorders and preventing tumorigenesis. Major advances towards understanding the mechanisms of flavones’ action in CID revealed their regulation of the key inflammatory pathways. Flavones affect these pathways either by changing protein–protein interactions or posttranslational modifications. However, how the flavones mechanistically orchestrate these effects needs further investigation. Towards that end, additional studies to identify direct molecular targets of flavones will be incredibly useful. Our own studies, screening protein libraries, revealed new unexpected mechanisms on how apigenin, through its direct interaction with an RNA binding protein, can regulate abnormal alternative splicing in cancer, results that lead to demonstrating how apigenin increases the efficacy of anti-cancer treatments [229,230,231]. The use of molecular dynamics has also provided valuable mechanistic insights into flavone interactions with proteins or DNA [232,233,234,235]. There are additional areas of unmet need that warrant further investigation including the development of food formulations that can deliver medically active doses and large epidemiological studies. Further research will provide a better understanding of the beneficial activities of flavones, which will help design preventive and therapeutic approaches for inflammatory diseases including obesity and cancer.

## Figures and Tables

**Table 2:** Molecular targets of flavones in obesity and cancer.

Target	Flavone	Dose	Activity	Model System	Ref.
		10, 30, 50 mg/kg/day *i.p.*	Systemic TNFα, IL-6, IL-1β, IL-12 ↓ *	C57Bl/6j male mice fed with HFD	[194]
		25 mg/kg/day *i.p.*	TNFα, IL-6, IFNγ, MCP-1 ↓	Pancreatic cancer in C57Bl/6j mice	[195]
	Apigenin	300 mg/kg/day *i.p*.	Nuclear NF-κB ↓	Human hepatocellular carcinoma xenografts in Balb/c nu/nu mice	[196]
		20 and 50 μg/mouse/day *p.o.*	Phospho-IκBα, IKKα, NF-κB p65 and p50, COX-2 ↓	Prostate cancer in C57Bl/6j mice	[197]
		40 μM	IKBKε, IL-1α, IL-6, MCP-1, GM-CSF ↓	Human MDA-MB-231 breast cancer cells	[198]
	Acacetin	3–100 μM	Phospho-IκB, nuclear p65, COX-2, TNFα, IL-6, MCP-1 ↓	Murine RAW264.7 macrophages cultured with 3T3-L1 adipocytes	[199]
	Baicalin	50 mg/kg/day *i.p*.	TNFα, MCP-1 mRNA ↓	WAT from HFD-fed C57Bl/6j male mice	[200]
	Baicalein	200, 400 mg/kg/day *p.o.*	NF-κB ↓	Murine melanoma B16-F10 xenografts in C57Bl/6j mice	[201]
NF-κB	Chrysin	25, 30 mg/kg/day *i.p.*	Systemic TNFα, IL-1β ↓	C57Bl/6j male mice fed with HFD	[202]
	Luteolin	0.005% w/w in HFD chow	Systemic TNFα, IL-1β, IL-6, MIP-1β ↓	C57Bl/6j male mice fed with HFD	[203]
		0.005% w/w in HFD chow	TNFα, IL-6, MCP-1 ↓	WAT from OVXed C57Bl/6j female mice fed with HFD *	[180]
	Nobiletin	10–100 μM	TNFα, MCP-1 ↓	Murine RAW264.7 macrophages cultured with 3T3-L1 adipocytes	[204]
	Tangeretin	20 mg/kg/day *p.o.*	TNFα, IL-6, IL-1β, MCP-1 ↓	WAT from HFD-fed C57Bl/6j male mice	[205]
		20, 40 μM	TNFα, IL-6, IL-1β, MCP-1 ↓	Murine bone marrow-derived macrophages cultured with adipocytes	[205]
	Vitexin	30, 60 mg/kg/day *p.o.*	Phospho-IκB, NF-κB p65, TNFα, IL-6, IL-1β ↓	C57Bl/6j male mice fed with HFD	[47]
		40, 160 mg/kg/day *p.o.*	Phospho-IκBα, nuclear p65, TNFα ↑	M1 macrophages from colon cancers in Balb/c mice	[206]
STAT	Apigenin	5–60 μM	Phospho-STAT1, IFNγ- induced PD-L1 expression ↓	Human melanoma A375, A2058, RPMI-7951 cell lines	[207]
		10–50 μM	Phospho-STAT1 and STAT3, PD-L1 expression ↓	Human NSCLC H460, H538, A549 cell lines	[208]
	Luteolin	50–100 μM	Phospho-JAK1 and STAT1, IL-8 ↓	Human colon cancer HT-29 cells	[209]
		0.3–10 μM	IL-6-induced STAT3 phosphorylation ↓	Human cholangiosarcoma KKU-M156 cells	[210]
Nrf2	Apigenin	1.56–6.25 μM	ARE-luciferase reporter, Nrf2-dependent gene HO-1 ↑	Human hepatocellular carcinoma HepG2 cells	[211]
	Nobiletin	10–100 μM	Nrf2-dependent gene HO-1 ↑	Murine RAW264.7 macrophages cultured with 3T3-L1 adipocytes	[204]
	Scutellarin	25, 50, 100 mg/kg/day *p.o.*	Nrf2 ↑ GSK, IL-1β, IL-2 ↓	Db/db diabetic mice	[212]
	Tangeretin	20–60 μM	Nrf2 ↓ Overcomes drug resistance	Human lung cancer A549/T cells	[213]
	Wogonin	20–60 μM	Nrf2 ↓ Overcomes drug resistance	Human breast cancer MCF7 cells	[214]
NLRP3		0.005% *w*/*w* in HFD chow	NLRP3, caspase-1, IL-1β ↓	WAT from OVXed C57Bl/6j female mice fed with HFD *	[215]
	Luteolin	50–150 μM	NLRP3, caspase-1, IL-1β ↑	Human colon cancer HT-29 cells	[216]
		50 mg/kg/day *i.p*.	NLRP3, caspase-1, IL-1β ↑	Human colon cancer cells xenografts in Balb/c nude mice	[216]

↓—decrease, ↑—increase, * OVXed—ovariectomized.

## Data Availability

Not applicable.
